# 

**Published:** 1863-11

**Authors:** 


### CONTENTS OF HALL'S JOURNAL OF HEALTH,

**FROM JANUARY TO SEPTEMBER, 1863.**

Farmer Health.
Eating.
Dress.
Shoes.
Corns.
Housewifery.
Potatoes.
Baldness.
Skating.
Premature Deaths.
Success in Life.
Mother's Influence.
Temper Controlled.
Our Daughters.
Unskilled Labor.
Wives Overtaxed.
Pulpit Power.
Witnesses Three.
Cherished Flower.
Temperance and Intemperance.
Wise Workers.
The Dying.
Industrial Facts.
Public Schools.
Cute Things.
Ventilation.
The One Spot.
Recreation.
Interesting Facts.
Reformers.
Randolph's Letter.
Bible Conformations.
Paine and Laurie Todd.
Sad Reflection.
Haunted House.
Physician and Patient.
Eyesight.
Surgical Diseases.
Burns.
Piles.
Philosophy.
Specifics.
One Acre.
Spring-Time.
Changing Clothing.
Eating Habits.
Fistulas.
Coffee Drinking.
Catching Cold.
Resignation.
Abuse of Medicine.
Major Sibley.
Constipation.
The Physician.
Pew System.
Emanations.
Drowning.
Dieting.
Diphtheria.
Loose Bowels.
Cholera.
Dysentery.
Bilious Diarrhea.
Disinfectants.
Physiologican.
Sabbath Observance.
Central Park.
Croup.
Making Money.
Escaping from Fire.
Saving Ministers.
Death Rate.
One by One.
Vices of Genius.
Leaving Home.
Fifth Avenue Sights.
Sickness Not Causeless.
Obscure Diseases.
Sayre the Banker.
Parental Corrections.
Raising Children.
Summer Hegira.
Worth Remembering.
Physiology of Worship.
Medical Items.
The Month Malign.
Great Eaters.
Logic Run Mad.
Insanity.
Philosophy of Exercise.
Summer Mortality.
Posture in Worship.

All subscriptions must begin with the January number, which begins the volume, making, at the end of the year, a valuable bound book of reference, with a copious index. When the loose numbers are returned, the bound volume is furnished by paying 25 cents for binding. Any back number of the JOURNAL, from Vol. I., No. 1, will be furnished for 10 cents.

### HALL'S JOURNAL OF HEALTH WITHOUT MONEY.

THE DOLLAR POCKET STEREOSCOPE will be sent, post-paid, for the Names and Subscription-Price of Two Subscribers for 1863. For Three Names, with Three Dollars, a Dollar and a Half Stereoscope will be sent; and for Six Names, with Six Dollars, a Three Dollar Stereoscope will be sent by mail, post-paid. These instruments are all the same in reality, except some are more ornamental than others.

TO THE EDITOR, the Pocket Stereoscope is a source of constant satisfaction and of quiet enjoyment—repeated as often as it is taken up—to look at the photographs of the members of his family, or absent friends, and at the Stereoscopic views of places visited abroad; because it so enlarges and brings out the pictures, as to make one almost believe that he is looking at the reality. You not only see the objects themselves, but the shade and portions of every view are brought to light which the naked eye fails to discover. This is not the case with any hand-painting, for it can not present what is invisible to the eye; but the sun-picture does, just as the telescope shows us worlds of light, which the unaided vision never could descry.

There is another source of pleasure in this Stereoscope. It is known that the tiniest objects in nature become more beautiful as they are magnified; art becomes coarser and less pleasing. Hence all photographic pictures are beautified by this charming little instrument. Hence it is suggested to

### OUR EXCHANGES, that they make arrangements, through us, with MR. GODFREY, of 831 BROADWAY, where these instruments are sold, to offer the Pocket Stereoscope as a premium for enlarging their subscriptions, as for this purpose it will be afforded on very liberal terms, and thus be the means of affording a large fund or source of quiet satisfaction and pleasure to such of their subscribers as choose to exercise a very little energy; a satisfaction which may be repeated every day during life, just as we never get tired of looking at nature, or at the faces of those we love; a source, too, of ceaselessly varying satisfaction; for the Stereoscope is about the size and shape of a common card-case, and by being carried in the pocket, can be used at the house of friends, in looking at their photographic albums and collections of other sun-pictures, single or double.

The Stereoscope does not strike all with the same force as it does ourselves; it requires some practice to get at its full use. Hence we do not advise any publisher to buy until he has tried them. He can then speak of them in proportion to his felt estimate of their value. We will send one to any of our exchanges, if 24 cents are sent to prepay postage. We want each one to be fully persuaded in his own mind, then nobody will be misled—"nobody hurt;" for there is no sort of use in attempting to palm any thing on the community unless it is of real worth—we mean, that it is of no advantage in the long run.

